# Preterm Birth Inequity—A Call for a Societal Movement

**DOI:** 10.1001/jamanetworkopen.2024.35855

**Published:** 2024-09-03

**Authors:** Heather H. Burris, Diana Montoya-Williams

**Affiliations:** Department of Pediatrics, University of Pennsylvania Perelman School of Medicine, Philadelphia; Department of Obstetrics and Gynecology, University of Pennsylvania Perelman School of Medicine, Philadelphia; Division of Neonatology, Children’s Hospital of Philadelphia, Philadelphia; Leonard Davis Institute of Health Economics, University of Pennsylvania, Philadelphia; Department of Pediatrics, University of Pennsylvania Perelman School of Medicine, Philadelphia; Division of Neonatology, Children’s Hospital of Philadelphia, Philadelphia; Leonard Davis Institute of Health Economics, University of Pennsylvania, Philadelphia; PolicyLab, Children’s Hospital of Philadelphia, Philadelphia, Pennsylvania

In their article, Jelliffe-Pawlowski et al^1^ report on preterm birth (PTB) rates in California among more than 5 million singleton births from 2011 to 2021 to evaluate temporal trends as well as racial and ethnic inequities. Specifically, the authors^1^ examined PTB rates within groups over time and explored potential risk and protective factors. Singleton PTB rates increased slightly over the study period, and substantial inequities persisted. In 2022, the highest PTB rates were among publicly insured individuals who identified as American Indian or Alaska Native (10.3%) and Black (11.3%).^1^ Historically, many US studies have compared PTB rates with a reference population (often White), which implies that there is something normal about the PTB rate among White individuals. That approach does not recognize the structural advantages that may reduce PTB risk for privileged individuals. We commend the authors’ choice to not present relative risks or odds ratios comparing racially and ethnically minoritized populations with White populations,^1^ which represents a methodologic advance. It refocuses the conversation on factors to promote equity by identifying structures and policies that are particularly harmful or helpful for disadvantaged groups. This is a necessary step to promote health justice.

The additional innovation in the study by Jelliffe-Pawlowski et al^1^ is the focus on solution-oriented, policy-relevant factors that may decrease PTB risk. Special Supplemental Nutrition Program for Women, Infants, and Children (WIC) participation was significantly associated with lower PTB risk among all groups of publicly insured individuals, but seemed to be associated with a larger risk reduction among American Indian or Alaska Native, Black, and Hispanic populations than Asian and White populations.^1^ Given the high rates of PTB among American Indian or Alaska Native and Black populations, one could envision enhanced and targeted public health messaging and resources focused on ensuring WIC provision for these groups, with the goal of mitigating existing perinatal health inequities. In addition, the group^1^ noted a steep rise in PTB rates among publicly insured Hispanic individuals, with higher rates among foreign-born compared with US-born Hispanic individuals. These findings challenge the well-documented Hispanic immigrant public health paradox in which, despite socioeconomic disadvantage, Hispanic immigrants have historically had better health outcomes than their US-born counterparts. Worsening PTB rates among Hispanic immigrants may be early evidence of the erosion of the Hispanic immigrant paradox that public health researchers have been worried about in light of the COVID-19 pandemic and other sociopolitical trends.^2^ Given recent signals of immigrant avoidance of health and social benefits like WIC,^3^ especially among Hispanic immigrants, this study’s findings^1^ may indicate that better public health messaging around WIC access is also needed for Hispanic communities as a strategy to mitigate the risk of emerging PTB inequities. Ultimately, identifying factors associated with higher and lower PTB risk, overall, within groups, and across time, displays the dynamic heterogeneity of PTB risk. For example, preexisting diabetes, hypertension, and housing insecurity were associated with higher risk of PTB for every group but risk ratios varied.^1^ This variation could be used to prioritize resource allocation for interventions to prevent PTB.

As noted by the authors,^1^ there are important next steps to improve perinatal health equity. PTB is not a single entity. There are many reasons that infants are born prior to 37 completed weeks of gestation. Spontaneous PTB (sPTB) occurs after rupture of membranes or preterm labor, while medically indicated PTB (mPTB) is clinician-initiated to optimize outcomes in the setting of conditions such as preeclampsia or impaired fetal growth. Risk factors for these conditions incompletely overlap, and distinct interventions would reduce their incidence. Ananth and colleagues^4,5^ have been reporting on preterm birth subtypes for decades. In the US, the proportion of singleton PTBs that are mPTBs has steadily risen from 26.0% in 1989 to 36.5% in 2000^4^ and to 41.1% in 2013.^5^ The classification of PTB as sPTB or mPTB was insufficient in the study by Jelliffe-Pawlowski et al^1^ to delineate time trends or risk factors for these distinct conditions given that 25.2% of PTBs were not able to be classified. Prioritization of public health resources to address PTB inequities requires a deeper understanding of the relative importance and documentation of sPTB and mPTB. A 2024 study^6^ in Philadelphia demonstrated a 20% wider disparity between Black and White individuals in mPTB than sPTB. Given its rise and major contribution to PTB inequities, mPTB reduction will be required to improve perinatal health equity.

Because hypertension and obesity are major risk factors for mPTB, a societal public health movement at multiple levels will be required to optimize the cardiometabolic health of girls, women, and birthing people prior to pregnancy to decrease mPTB risk and its racial and economic inequities. Additionally, multisectoral work is needed to ensure people access high-quality health care before and during pregnancy that is responsive to their unique needs, especially when sociopolitical dynamics enhance or create threats to access, quality, and trust. In their call for “Reparations as a Public Health Priority,” Bassett et al^7^ argue that investments to reduce resource gaps, stress, and improve intergenerational health in racially and ethnically minoritized and disadvantaged communities and families could improve health outcomes. Today not every child has the opportunity to breathe clean air, drink uncontaminated water, play outside in safe and green spaces, access a nutritious diet, learn in thriving schools, and receive medical attention in high-quality health care systems. Children from racially and ethnically minoritized and low-income families are less likely to have these opportunities that can improve the chances of optimal health leading into pregnancy. Large-scale redistribution of wealth in the US is likely necessary to realize the health-promoting social and physical environments required for health equity across the lifespan.
